# Long-Lasting Desynchronization of Plastic Neural Networks by Random Reset Stimulation

**DOI:** 10.3389/fphys.2020.622620

**Published:** 2021-02-05

**Authors:** Ali Khaledi-Nasab, Justus A. Kromer, Peter A. Tass

**Affiliations:** Department of Neurosurgery, Stanford University, Stanford, CA, United States

**Keywords:** random reset stimulation, spike-timing dependent plasticity (STDP), desynchronization, segmented electrodes, long-lasting effects

## Abstract

Excessive neuronal synchrony is a hallmark of neurological disorders such as epilepsy and Parkinson's disease. An established treatment for medically refractory Parkinson's disease is high-frequency (HF) deep brain stimulation (DBS). However, symptoms return shortly after cessation of HF-DBS. Recently developed decoupling stimulation approaches, such as Random Reset (RR) stimulation, specifically target pathological connections to achieve long-lasting desynchronization. During RR stimulation, a temporally and spatially randomized stimulus pattern is administered. However, spatial randomization, as presented so far, may be difficult to realize in a DBS-like setup due to insufficient spatial resolution. Motivated by recently developed segmented DBS electrodes with multiple stimulation sites, we present a RR stimulation protocol that copes with the limited spatial resolution of currently available depth electrodes for DBS. Specifically, spatial randomization is realized by delivering stimuli simultaneously to *L* randomly selected stimulation sites out of a total of *M* stimulation sites, which will be called L/M-RR stimulation. We study decoupling by L/M-RR stimulation in networks of excitatory integrate-and-fire neurons with spike-timing dependent plasticity by means of theoretical and computational analysis. We find that L/M-RR stimulation yields parameter-robust decoupling and long-lasting desynchronization. Furthermore, our theory reveals that strong high-frequency stimulation is not suitable for inducing long-lasting desynchronization effects. As a consequence, low and high frequency L/M-RR stimulation affect synaptic weights in qualitatively different ways. Our simulations confirm these predictions and show that qualitative differences between low and high frequency L/M-RR stimulation are present across a wide range of stimulation parameters, rendering stimulation with intermediate frequencies most efficient. Remarkably, we find that L/M-RR stimulation does not rely on a high spatial resolution, characterized by the density of stimulation sites in a target area, corresponding to a large *M*. In fact, L/M-RR stimulation with low resolution performs even better at low stimulation amplitudes. Our results provide computational evidence that L/M-RR stimulation may present a way to exploit modern segmented lead electrodes for long-lasting therapeutic effects.

## 1. Introduction

Synchronization of coupled oscillators is observed in various fields of the natural sciences, for instance, in neurosciences (Steriade et al., 1990; Haken, 2006), medicine (Tass, 1999), physics (Pikovsky et al., 2001; Haken, 2012), biology (Winfree, 2001), and chemistry (Kuramoto, 2003). In the nervous system, synchronization of neuronal activity is critical for successful motor control (Andres and Gerloff, 1999) and information processing (Singer, 1990). However, excessive synchronization is associated with several neurological disorders, e.g., essential tremor, Parkinson's disease (PD) (Alberts et al., 1969; Nini et al., 1995), epilepsy (Mormann et al., 2000), and chronic subjective tinnitus (Eggermont and Tass, 2015).

High-frequency deep brain stimulation (HF DBS) is the standard of care for patients with advanced PD. HF DBS is delivered to target brain regions, such as the subthalamic nucleus (STN), through implanted lead electrodes. The mechanism of action of DBS is still a matter of debate (Ashkan et al., 2017; Jakobs et al., 2019; Lozano et al., 2019). As PD symptoms return shortly after cessation of stimulation, permanent delivery of HF DBS is required for persistent symptom suppression (Temperli et al., 2003). On the other hand, permanent stimulation increases the risk of side effects such as depression, cognitive decline, speech difficulty, instability, dyskinesia, and gait disorders (Rodriguez-Oroz et al., 2005; Temel et al., 2006). The risk of unwanted side effects may be reduced by a substantial reduction of the delivered stimulation current.

Early studies on desynchronization focused on single pulses delivered to a vulnerable phase of a collective oscillation (Mines, 1914; Winfree, 1977, 1980; Warman and Durand, 1989; Tass, 1999), followed by the development of composite single-channel (Tass, 2001, 2002; Zhai et al., 2005) and multi-channel stimuli (Tass, 2003) to further improve the robustness of the desynchronizing effects. In addition, linear or non-linear delayed feedback was used to desynchronize model networks (Rosenblum and Pikovsky, 2004a,b; Hauptmann et al., 2005a,b,c; Popovych et al., 2005, 2006a,b; Pyragas et al., 2007; Popovych and Tass, 2010). The latter approaches might clinically be applied by using the linear or non-linear delayed feedback as an envelope of pulse trains (Popovych et al., 2017a,b). By estimating phase response curves, researchers also identified well-tuned periodic stimulation (Wilson et al., 2011), and the delivery of well-timed stimulation bursts as possible desynchronizing stimulation approaches (Holt et al., 2016). A desynchronization approach that does not rely on well-timed stimuli is coordinated reset (CR) stimulation (Tass, 2003). During CR stimulation, desynchronization is achieved by delivering phase-shifted stimuli to multiple subpopulations of oscillators.

Originally, the mentioned desynchronization techniques have been developed for networks of oscillators with fixed connection strengths. In the brain, however, neuronal networks are subject to synaptic plasticity that alters synaptic weights according to neuronal activity. A prominent mechanism leading to adaptive connectivity is spike-timing dependent plasticity (STDP), which modifies the coupling strengths based on the relative timing of post- and presynaptic spikes (Markram et al., 1997; Abbott and Nelson, 2000; Caporale and Dan, 2008). In several brain areas, STDP strengthens synapses if the postsynaptic neuron fires shortly after the presynaptic one, otherwise the connections become weaker (Markram et al., 1997; Bi and Poo, 1998). Plasticity mechanisms can stabilize certain activity patterns, such as synchronized activity (Karbowski and Ermentrout, 2002) and may lead to the formation of strongly connected neuronal assemblies (Litwin-Kumar and Doiron, 2014). A recent study showed that in presence of STDP, self-organized clusters could emerge, whereby the clusters divide the networks into groups that are synchronized at different firing frequencies (Röhr et al., 2019). Furthermore, the interplay of network adaptation and collective spiking activity can lead to the coexistence of distinct stable states, such as synchronized, desynchronized, and cluster states (Seliger et al., 2002; Zanette and Mikhailov, 2004; Tass and Majtanik, 2006; Maistrenko et al., 2007; Masuda and Kori, 2007; Aoki and Aoyagi, 2009; Röhr et al., 2019; Berner et al., 2020; Yanchuk et al., 2020).

Stimulation-induced changes of synaptic connectivity may drive the network into an attractor of a stable desynchronized state and cause long-lasting desynchronization (Tass and Majtanik, 2006). Such long-lasting desynchronization may follow after coordinated reset stimulation as shown by extensive theoretical (Tass and Majtanik, 2006; Kromer and Tass, 2020; Kromer et al., 2020) and computational studies (Tass and Majtanik, 2006; Hauptmann and Tass, 2009; Popovych and Tass, 2012; Lourens et al., 2015; Manos et al., 2018). Corresponding long-lasting desynchronization and therapeutic effects have been confirmed experimentally (Tass et al., 2009), as well as in preclinical (Adamchic et al., 2014; Wang et al., 2016) and clinical studies (Adamchic et al., 2014).

In preclinical and clinical studies, the frequency of CR stimulation has been adjusted to the frequency of the synchronous rhythm as measured by the dominant peak in the power spectrum of the local field potential (Tass et al., 2012; Adamchic et al., 2014; Wang et al., 2016). This parameter choice is motivated by the original idea of CR stimulation; to excite higher-order modes of the Kuramoto order parameter (Tass, 2003). Additionally, recent computational studies indicate that long-lasting desynchronization effects of CR stimulation are more pronounced for well-adjusted CR frequencies (Manos et al., 2018; Kromer and Tass, 2020; Kromer et al., 2020), this includes adjusting the stimulation frequency to the dominant neuronal rhythm (Tass, 2003; Adamchic et al., 2014; Manos et al., 2018) or the STDP time scale (Kromer et al., 2020). This may limit the clinical applicability of CR stimulation as a treatment for PD as different symptoms are associated with pathological synchrony in different frequency bands. In more detail, excessive synchronization of basal ganglia activity in the theta band (3−10 Hz) is associated with symptoms such as dyskinesia and tremor (Brown, 2003; Steigerwald et al., 2008; Tass et al., 2010; Contarino et al., 2012), while synchronized activity in the beta band (13−30 Hz) is associated with rigidity and bradykinesia (Kühn et al., 2006; Weinberger et al., 2006).

In order to increase the robustness of long-lasting effects with respect to stimulation parameters, such as the stimulation frequency, Kromer and Tass (2020) suggested a Random Reset (RR) stimulation protocol. RR refers to the delivery of stimuli in a temporally and spatially randomized manner. In their theoretical work, temporal randomization was realized by delivering stimuli at random times, with exponentially distributed interstimulus intervals. Thus, stimulation times followed a Poisson spike train. Spatial randomization was realized by randomly selecting 50% of the neurons for stimulus application at each stimulation time—irrespective of the neurons' locations relative to realistic spatial stimulation profiles. This implicitly assumed “microscopic control,” i.e., that even nearby neurons can be stimulated independently, which is not possible in DBS-like setups. Remarkably, the suggested RR stimulation method led to robust, long-lasting desynchronization effects after stimulation ceases even though the neurons remained partially synchronized during the entire stimulation period. This was achieved by a pronounced stimulation-induced decoupling of the neurons. Therefore, decoupling stimulation was suggested as the primary goal in order to weaken synaptic connections rather than counteracting synchronization as in previous approaches (Kromer and Tass, 2020).

Segmented depth electrodes for DBS enable spatially selective steering of stimulation current (Krack et al., 2003; Buhlmann et al., 2011; Steigerwald et al., 2019; Krauss et al., 2020). However, so far, a pressing question regarding a possible implementation of RR stimulation using available DBS electrodes is that to which extent the observed decoupling effects rely on the spatial randomization. In particular, whether it is really necessary to deliver randomly timed stimuli to individual neurons or whether delivery to macroscopic neuronal subpopulations is sufficient. Experimentally using segmented DBS electrodes that allow for independent activation of multiple stimulation contacts, one can deliver stimuli to neuronal subpopulations. Traditionally DBS is delivered through a flexible cylinder with 4 stimulation contacts (Gielen, 2001; Butson and McIntyre, 2005). In order to deliver stimuli to individual neuronal subpopulations, modern electrodes are capable of directional current steering (Buhlmann et al., 2011; Steigerwald et al., 2019; Krauss et al., 2020)

Directional steering allows for realizing spatiotemporal current profiles by superposition of stimuli delivered to individual stimulation contacts (Buhlmann et al., 2011; Steigerwald et al., 2019). To improve spatial selectivity, recent research in electrode design is devoted to segmented multisite electrodes with increasing numbers of stimulation contacts (Buhlmann et al., 2011; Steigerwald et al., 2019; Krauss et al., 2020). For instance, certain designs allow for selective activation of up to 32 stimulation contacts (Contarino et al., 2014; Steigerwald et al., 2019).

In the present paper, we study a new implementation of RR stimulation using available DBS electrodes, where we introduce a version of RR stimulation in which individual stimuli are simultaneously delivered to *L* out of *M* randomly selected spatially coherent neuronal subpopulations, here called L/M-RR stimulation. This approach only requires “mesoscopic control,” i.e., independent stimulation of neuronal subpopulations, and not “microscopic control,” i.e., independent stimulation of single neurons, as in the approach of Kromer and Tass (2020). For our analysis, we use a combination of theoretical predictions and simulations of networks of leaky integrate-and-fire (LIF) neurons with STDP. While our theory predicts efficient decoupling for a wide range of stimulation parameters, it reveals qualitative differences between low and high-frequency L/M-RR stimulation, section 3.1. While low-frequency L/M-RR stimulation yields parameter robust decoupling and related long-lasting effects, the performance of high-frequency L/M-RR stimulation is limited to small numbers of simultaneously stimulated subpopulations. These qualitative differences are present for a wide range of stimulation amplitudes and render strong high-frequency L/M-RR stimulation ineffective in terms of long-lasting after-effects, see section 3.2. Finally, in section 3.3, we analyze how the size of individual subpopulations influence the long-lasting effects. Remarkably, we find that stimulation of large subpopulations yields better results for weak stimulation, see section 3.3. Simultaneous stimulation of large neuronal subpopulations may hence be advantageous for possible realizations of L/M-RR for DBS.

## 2. Models and Methods

### 2.1. Neuronal Network Model

We consider a network of *N* conductance-based LIF neurons with STDP previously presented in Kromer et al. (2020). Throughout the paper, we fix the network size to *N* = 1, 000. Neurons are organized along the *x*-axis. Individual neurons' center locations *x*_*i*_ are uniformly distributed in the interval *x*_*i*_ ∈ [−2.5, 2.5] mm, which is motivated by the width used in an ellipsoidal volume approximation of the STN in detailed computational studies of STN DBS (Ebert et al., 2014). Random excitatory synaptic connections are added such that the total connectivity is 7%. The probability for two neurons to form a synaptic connection depends on the distance between the neurons as *p*∝exp((|*x*_*j*_ − *x*_*i*_|)/0.5mm) (Ebert et al., 2014).

The subthreshold dynamics of the membrane potential *v*_*i*_ of neuron *i* obeys

(1) $$\begin{array}{l} C_{i}\frac{\text{d}v_{i}}{\text{d}t}=g_{\text{leak}}\left(v_{\text{rest}}-v_{i}\right)+I_{i}^{\text{syn}}\left(t\right)+S_{i}\left(t\right)+I_{i}^{\text{noise}}\left(t\right). \end{array}$$

*C*_*i*_ is the membrane capacitance, *v*_rest_ the resting potential, *g*_leak_ = 0.02 mS/cm^2^ the leakage conductance, $I_{i}^{\text{syn}}\left(t\right)$ the synaptic input current, *S*_*i*_(*t*) the stimulation current, and $I_{i}^{\text{noise}}\left(t\right)$ the noisy input current.

Spiking occurs when *v*_*i*_ crosses a dynamic threshold potential $v_{i}^{\text{th}}$ given by

(2) $$\begin{array}{l} \tau_{\text{th}}\frac{\text{d}v_{i}^{\text{th}}}{\text{d}t}=\left(v_{\text{rest}}^{\text{th}}-v_{i}^{\text{th}}\right). \end{array}$$

Here, τ_th_ is the threshold time constant and $v_{\text{rest}}^{\text{th}}$ the stationary threshold potential. Artificial spikes are realized by setting the membrane potential *v*_*i*_ → *v*_spike_ for a time period of *t*_spike_ after a threshold crossing. Afterwards, a reset is performed by setting *v*_*i*_ → *v*_reset_ and $v_{i}^{\text{th}}\to v_{\text{spike}}^{\text{th}}$.

Throughout the paper, we use the same parameters as in Kromer et al. (2020): *v*_rest_ = −38 mV, $v_{\text{rest}}^{\text{th}}=-40$ mV, *t*_spike_ = 1 ms, τ_th_ = 5 ms, *v*_spike_ = 20 mV, *V*_reset_ = −67 mV. The *C*_*i*_'s follow a normal distribution with a mean value of 〈*C*_*i*_〉 = 3 μF/cm^2^ and a standard deviation of 0.05〈*C*_*i*_〉.

Excitatory synaptic input $I_{i}^{\text{syn}}\left(t\right)$ to neuron *i*, is given by

(3) $$\begin{array}{l} I_{i}^{\text{syn}}=g_{i}^{\text{syn}}\left(v_{\text{syn}}-v_{i}\right), \end{array}$$

$$\begin{array}{l} \tau_{\text{syn}}\frac{\text{d}g_{i}^{\text{syn}}}{\text{d}t}=-g_{i}^{\text{syn}}+\frac{\kappa }{N}\underset{j\in G_{i}}{\sum }w_{ji}\underset{l^{j}}{\sum }\delta \left(t-t_{l^{j}}^{j}-t_{\text{d}}\right), \end{array}$$

where $g_{i}^{\text{syn}}$ is the synaptic conductance, κ = 8 mS/cm^2^ is the coupling strength, *v*_syn_ = 0 mV is the synaptic reversal potential, τ_syn_ = 1 ms the synaptic time scale, *w*_*ji*_ ∈ [0, 1] is the synaptic weight between presynaptic neuron *j* and postsynaptic neuron *i*. The first sum runs over all presynaptic neurons, and the second sum runs over the spikes of the presynaptic neuron *j*. *G*_*i*_ is the set of indices of all presynaptic neurons to neuron *i*. $t_{l_{j}}^{j}$ is the *l*_*j*_th spike time of neuron *j*. We consider homogeneous synaptic delays of *t*_d_ = 3 ms.

In addition to presynaptic input from other neurons in the network, each neuron *i* receives noisy input, e.g., from other brain regions. The resulting input current $I_{i}^{\text{noise}}$ is obtained by feeding independent presynaptic Poisson spike trains with firing rate *f*_noise_ = 20 Hz into excitatory synapses on each neuron *i* (Ebert et al., 2014)

(4) $$\begin{array}{l} I_{i}^{\text{noise}}=g_{i}^{\text{noise}}\left(v_{\text{syn}}-v_{i}\right), \end{array}$$

$$\begin{array}{l} \tau_{\text{syn}}\frac{\text{d}g_{i}^{\text{noise}}}{\text{d}t}=-g_{i}^{\text{noise}}+D\tau_{syn}\underset{k_{i}}{\sum }\delta \left(t_{k_{i}}^{i}-t\right). \end{array}$$

Where $g_{i}^{\text{noise}}\left(t\right)$ is the synaptic conductance, τ_syn_ = 1 ms, and *v*_syn_ = 0 mV. The noise intensity is controlled by the parameter *D* = 0.026 *mS*/*cm*^2^ scaling the strength of the Poisson input.

### 2.2. Spike-Timing Dependent Plasticity (STDP)

During ongoing spiking, synaptic weights *w*_*ij*_ evolve according to a nearest-neighbor STDP scheme (Burkitt et al., 2004). Following previous studies on CR stimulation (Popovych and Tass, 2012), we consider a scheme where each arrival of a presynaptic spike at a postsynaptic neuron *j* (at time *t* = *t*_*i*_ + *t*_d_) and each postsynaptic spike (at time *t* = *t*_*j*_) cause an update of the synaptic weight, i.e., *w*_*ij*_ → *w*_*ij*_ + *W*(*t*_*j*_ − (*t*_*i*_ + *t*_d_)). Here, *t*_*i*_ denotes the spike time of the presynaptic spike. *W*(*t*) is the STDP function and is given by two exponentials (Song et al., 2000; Kromer and Tass, 2020)

(5) $$W(\Delta t)=\eta \{\begin{array}{ll} e^{-|\Delta t|/\tau_{+}}, & \Delta t>0\\ 0, & \Delta t=0\\ -\frac{\beta }{\tau_{R}}\;e^{-|\Delta t|/\tau_{-}} & \Delta t<0. \end{array}$$

Δ*t* = *t*_*j*_ − (*t*_*i*_ + *t*_d_) is the time lag between postsynaptic spike times and presynaptic spike arrival times. η = 0.02 scales the weight update per spike, τ_+_ = 10 ms and τ_−_ = τ_+_τ_*R*_ are the STDP decay times for long-term potentiation (LTP) and long-term depression (LTD), respectively, τ_*R*_ = 4 yields asymmetry in STDP decay times, and β = 1.4 scales the ratio of overall LTD to LTP. These STDP parameters lead to bistability between a strongly connected state with synchronized neuronal activity and a weakly connected state with asynchronous neuronal activity (Kromer and Tass, 2020; Kromer et al., 2020).

### 2.3. Quantification of Synchronization

In order to quantify the degree of in-phase synchronization, we calculate the time-averaged Kuramoto order parameter (Kuramoto, 1984)

(6) $$\rho_{\Delta }(t)=\frac{1}{\Delta }\int_{t-\frac{\Delta }{2}}^{t+\frac{\Delta }{2}}dt^{\prime }\left|\frac{1}{N}\sum_{k=0}^{N-1}e^{-i\psi_{k}(t^{\prime })}\right|.$$

Here, ψ_*k*_(*t*) is a phase function that increases linearly in time during individual interspike intervals of neuron *k*, i.e., $\psi_{k}\left(t\right)=2\pi \left(\left(t-t_{l}^{k}\right)/\left(t_{l+1}^{k}-t_{l}^{k}\right)+l\right)$ for $t\in \left[t_{l}^{k},t_{l+1}^{k}\right)$ (Rosenblum et al., 2001). ρ_Δ_ quantifies the degree of synchronization of a population of *N* neurons during the time interval Δ = 10 s. Perfect in-phase synchronization results in ρ_Δ_ = 1 whereas ρ_Δ_ ≈ 0 refers to absence of in-phase synchronized neuronal activity.

### 2.4. L/M-Random Reset Stimulation

Throughout the paper, we deliver RR stimulation to a randomly selected group of neuronal subpopulations. RR stimulation is characterized by the delivery of temporally and spatially randomized stimulus patterns. Temporal randomization is realized by delivering stimuli at random times *s*_*k*_. Interstimulus intervals *S*_*k*_ = *s*_*k*+1_ − *s*_*k*_ are distributed according to an exponential distribution with minimum interstimulus interval τ_Λ_ (Kromer and Tass, 2020)

(7) $$P(S_{k})\propto \exp(-\frac{S_{k}-\tau_{\Lambda }}{\tau_{\text{RR}}})\Theta (S_{k}-\tau_{\Lambda }).$$

Where τ_Λ_ = 1/130 s is the minimum interstimulus interval which corresponds to a maximal stimulation frequency of 130 Hz. This frequency is often used in clinical DBS studies (Krauss et al., 2020).

**Figure 1:**
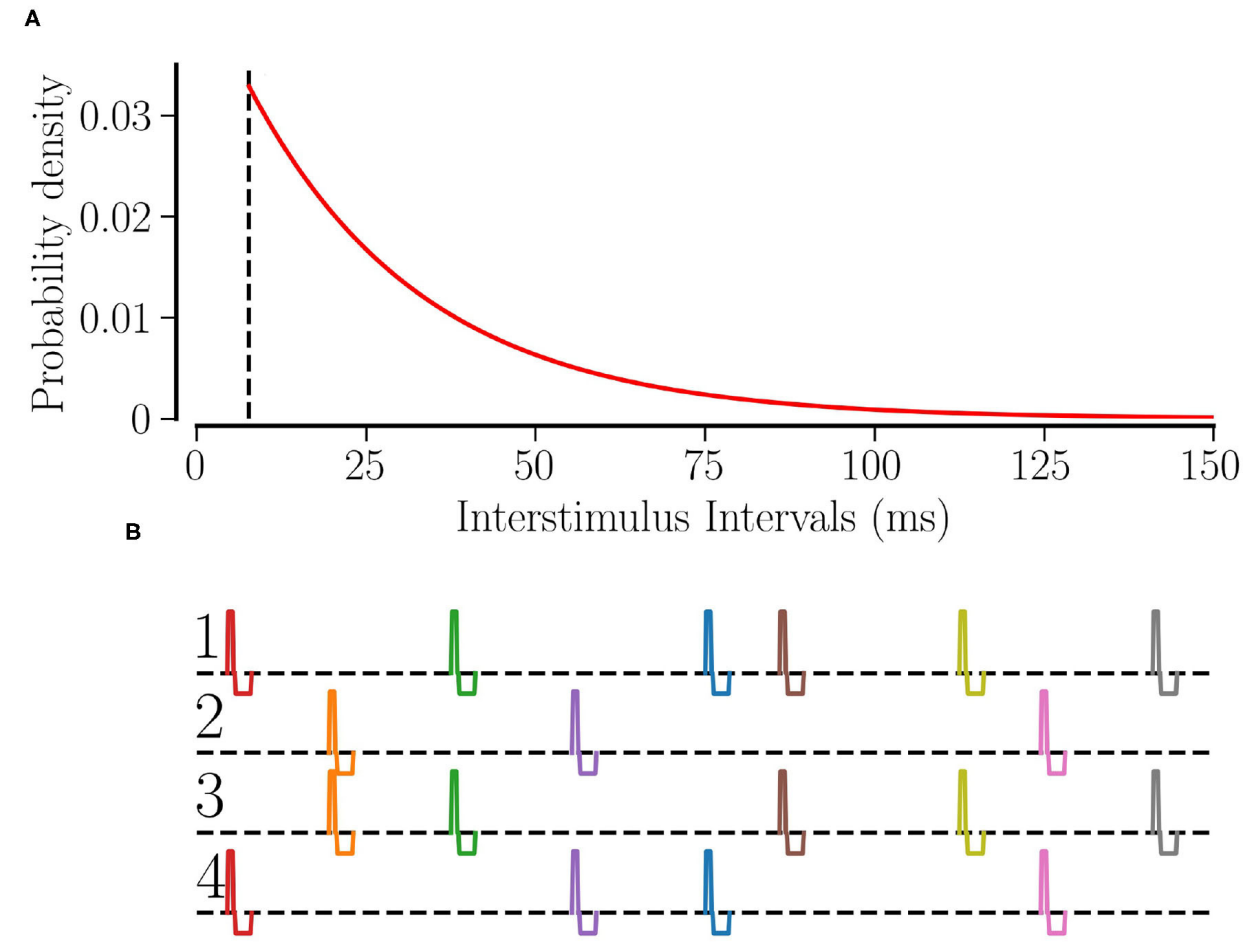
RR stimulation: **(A)** the probability distribution of interstimulus intervals for *f*_RR_ = 30 Hz. The dashed line shows the minimum interstimulus interval, $\tau_{\Lambda }={\left(130\;\text{Hz}\right)}^{-1}$. **(B)** The schematic of RR stimulation for *M* = 4 total sites where 2 sites are activated simultaneously at random times. The excitatory pulse has a duration of ν_e_ = 0.5 ms and an amplitude of $A_{\text{e}}=A_{\text{stim}}\mu /\nu_{\text{e}}$; separated by a gap of 0.2 ms, the inhibitory pulse has a duration of ν_i_ = 1.5 ms and an amplitude of $A_{\text{i}}=A_{\text{stim}}\mu /\nu_{\text{i}}$.

Θ(*t*) is the Heaviside step function, τ_RR_ determines the average stimulation frequency *f*_RR_ by *f*_RR_: = 1/(τ_Λ_ + τ_RR_). Figure 1A shows the distribution of interstimulus intervals for *f*_RR_ = 30 Hz.

**Figure 2:**
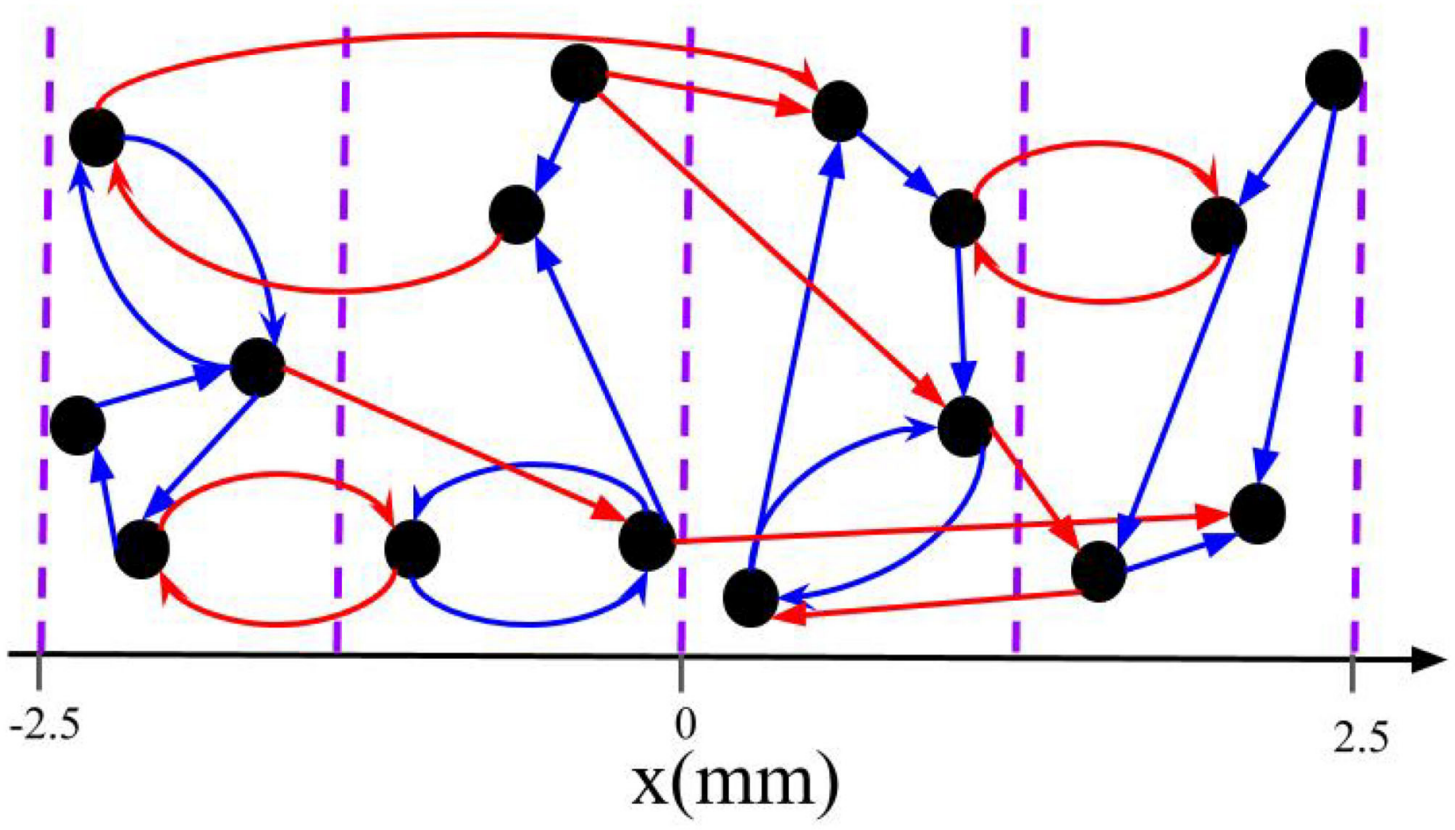
Schematic of intrapopulation and interpopulation synapses for a total of *M* = *4* subpopulations. The blue arrows represent the intrapopulation synapses where both pre- and postsynaptic neurons are within the same group. The red arrows show the interpopulation synapses where the pre- and postsynaptic neurons are in different subpopulations. Note that neurons are distributed along the *x*-axis and the vertical axis is merely symbolic.

In the present study, spatial randomization is realized by delivering each stimulus (at time *s*_*k*_) to a randomly selected group of *L* out of *M* neuronal subpopulations. Neurons are assigned to subpopulations according to their centers' locations. In particular, neuron *i* is considered to be part of subpopulation *l* if $x_{i}\in \left[-2.5+\frac{5\left(l-1\right)}{M},-2.5+\frac{5l}{M}\right)$ mm, *l* = 1, 2, ..., *M*. Hence, *M* scales the required spatial resolution. This setup is motivated by the shape of commonly used cylindrical DBS electrodes with equidistantly placed stimulation contacts (Gielen, 2001; Krauss et al., 2020). Figure 2 illustrates the division into subpopulations for *M* = 4 sites. At each stimulation time *s*_*k*_, a stimulus is delivered to *L* out of *M* randomly selected subpopulations without replacement. Thus, at each stimulation time the first electrode is selected uniformly at random with a probability of $\frac{1}{M}$, and for the second one we select one of the remaining electrodes with the probability of $\frac{1}{M-1}$ and so on. If not stated otherwise, we use *M* = 32 throughout the paper. This mimics a recently developed DBS lead electrode with up to 32 stimulation contacts that can be activated independently (Steigerwald et al., 2016).

Individual stimuli are charge-balanced and consist of an excitatory and an inhibitory rectangular pulse. The excitatory pulse has a duration of ν_e_ = 0.5 ms and an amplitude of $A_{\text{e}}=A_{\text{stim}}\mu /\nu_{\text{e}}$; separated by a gap of 0.2 ms, the inhibitory pulse has a duration of ν_i_ = 1.5 ms and an amplitude of $A_{\text{i}}=-A_{\text{stim}}\mu /\nu_{\text{i}}$ where μ = (*V*_th,spike_ − *V*_reset_)/〈*C*_*i*_〉. Thus, a stimulus of stimulation strength *A*_stim_ = 1 will elevate the membrane potential above the spiking threshold no matter when the last spike of the neuron occurred. Figure 1B shows a schematic of our L/M-RR stimulation protocol for the case where each stimulus is delivered to *L* = 2 (out of *M* = 4) stimulation sites.

### 2.5. Calculation of Mean Rate of Weight Change

The development of RR stimulation was originally based on theoretical predictions of the stimulation-induced synaptic weight dynamics during randomized stimulation. The corresponding theoretical study can be found in Kromer and Tass (2020). In the following, we briefly state the main steps and expand their results to our L/M-RR stimulation protocol.

The approach of Kromer and Tass (2020) and others (Kempter et al., 1999; Burkitt et al., 2004) is based on the mean rate of weight change $J_{ij}$ for a single synaptic weight *w*_*ij*_ with presynaptic neuron *i* and postsynaptic neuron *j*. Averaging over a long time interval and a large number of realizations of the spike train, and assuming that post- and presynaptic neurons have the same mean firing rate *r*, the mean rate of weight change can be written as

(8) $$\begin{array}{l} \langle J_{ij}\rangle \approx r\overset{\infty }{\underset{-\infty }{\int }}dt^{\prime }\;G_{ij}\left(t^{\prime }\right)W\left(t^{\prime }-t_{d}\right). \end{array}$$

For the case of different firing rates of neurons *i* and *j*, we refer to Kromer and Tass (2020). Here *G*_*ij*_(*t*) is the average number of time lags *t* per spike in the infinitesimal interval *t* ∈ [*t, t* + *dt*) that contribute to weight updates for the chosen STDP scheme. Note that *G*_*ij*_(*t*) is in general not normalized to one, but to the mean number of time lags per spike that contribute to weight updates.

Kromer and Tass (2020) derived results for *G*_*ij*_(*t*) in the limit of strong and fast stimulation, where each stimulus triggers a spike, and all spikes are triggered by stimuli. Here *strong* refers to large stimulation amplitudes *A*_stim_ ≈ 1 and *fast* to stimulation frequencies that are large compared to the firing rate in the synchronous state.

In the limit of strong and fast stimulation, the neurons' firing rates equal the frequency of stimulus administration *r*_*i*_ = *r*_*j*_ = *f*_RR_*L*/*M*, and the distribution of time lags that lead to weight updates due to STDP results from the distribution of interstimulus intervals between stimuli delivered to the post- and presynaptic neurons, respectively (Kromer and Tass, 2020). We distinguish between two contributions to these time lags *t* by setting *t* = *S* + ξ. Here, *S* is the difference between post- and presynaptic stimulation times and ξ characterizes the difference in delayed spiking responses to stimuli. Note that only time lags that lead to weight updates are considered. Therefore, *S* does not denote any interstimulus interval between stimuli delivered to the post- and presynaptic neurons, but only those for which the time lag between the triggered post- and presynaptic spike results in a weight update. Then, *G*_*ij*_(*t*) results from the distributions of ξ and *S*. First, we consider the distribution of ξ. In the limit of strong and fast stimulation, each stimulus triggers a spike of a stimulated neuron and each spike is triggered by a stimulus. We assume that the time lag *t*′ between stimulus delivery and spiking response of the stimulated neuron is distributed according to a distribution λ(*t*′). Further, assuming that spiking responses of the pre- and postsynaptic neuron follow this distribution, we find that ξ is distributed according to $Z\left(\xi \right)=\int_{-\infty }^{\infty }dt^{\prime }\;\lambda \left(t^{\prime }\right)\lambda \left(t^{\prime }+\xi \right)$. Second, we denote the distribution of *S* as *p*_*ij*_(*S*, ξ). Note that, in general, *p*_*ij*_(*S*, ξ) depends on the realization of ξ. This is because certain realizations of ξ may change the order of spike and spike arrival times of the pre- and postsynaptic neurons' spikes. The latter may affect which time lags contribute to weight updates, and therefore, which interstimulus intervals *S* need to be considered.

In order to calculate *p*_*ij*_(*S*, ξ), we follow the approach of Kromer and Tass (2020) and consider possible pairings of a presynaptic (postsynaptic) spike that is triggered by the *n*th stimulus with postsynaptic (presynaptic) spikes. We distinguish between two scenarios. In the first scenario, both post- and presynaptic neuron are stimulated simultaneously, while in the second one either the postsynaptic or the presynaptic neuron receives the *n*th stimulus.

In Kromer and Tass (2020) results for *p*_*ij*_(*S*, ξ) for either case were derived. In the first case, the distribution of interstimulus intervals is given by

(9) $$\begin{array}{l} p_{ij}^{\text{I}}\left(S|\xi ≶t_{d}\right)=\delta \left(S\right)+F\left(\pm S\right) \end{array}$$

with

(10) $$\begin{array}{l} F\left(S\right)=\overset{\infty }{\underset{k=1}{\sum }}\frac{L}{M}{\left(1-\frac{L}{M}\right)}^{k-1}P*....*P\left(S\right). \end{array}$$

Here the *k*th summand contains the (*k* − 1)th convolution with the zeroth convolution referring to *P*(*S*) itself. The latter is given in Equation (7). δ(*x*) denotes the Dirac delta distribution.

As can be seen in Equation (9), the realization of ξ determines which interstimulus intervals *S* are considered for weight updates resulting from the current presynaptic spiking event. If ξ is larger than the synaptic delay *t*_*d*_ the presynaptic spike arrives before the postsynaptic spike and the arrival time is paired with the current postsynaptic spike (positive update) and the latest postsynaptic spike, triggered by an earlier stimulus delivered to the postsynaptic neuron (negative update). *F*(−*S*) denotes the probability that the latest stimulus was delivered to the postsynaptic neuron with an interstimulus interval *S* relative to the current stimulus. In contrast, if ξ is smaller than the delay time, the presynaptic spike arrives after the postsynaptic one and its arrival time is paired with the current postsynaptic spike (negative update) and the next postsynaptic spike that results from the next stimulus delivered to the postsynaptic neuron (positive update).

In the second case, *p*_*ij*_(*S*, ξ) does not depend on ξ, if synaptic delays are short compared to interstimulus intervals and λ(*t*) is narrow compared to interstimulus intervals. *p*_*ij*_(*S*, ξ) given by

(11) $$\begin{array}{l} p_{ij}^{\text{II}}\left(S,\xi \right)=p_{ij}^{\text{II}}\left(S\right)=F\left(S\right)+F\left(-S\right). \end{array}$$

Here, the current presynaptic spike arrival time is paired with postsynaptic spikes triggered by the latest (negative update) and the next stimulus delivered to the postsynaptic neuron (positive update), respectively.

Using Equations (9), (11), and (10), the distribution of time lags *G*_*ij*_(*t*) results from

(12) $$\begin{array}{l} G_{ij}^{\text{I/II}}\left(t\right)=\int ds\;Z\left(t-s\right)p_{ij}^{\text{I/II}}\left(s,t-s\right). \end{array}$$

We separate synapses into two groups based on the probability of receiving stimuli simultaneously during L/M-RR stimulation. The first group consists of synapses that connect neurons within the same subpopulation. This group will be referred to as *intrapopulation* synapses. The second group consists of synapses between neurons belonging to different subpopulations. These synapses will be referred to as *interpopulation* synapses. The classification of intra- and interpopulation synapses is illustrated for a toy network in Figure 2.

First, we consider intrapopulation synapses. Corresponding quantities will be marked by the suffix “intra” in the following. Neurons connected by intrapopulation synapses always receive stimuli simultaneously. In consequence, the distribution of time lags is given by

(13) $$\begin{array}{l} G^{\text{intra}}\left(t\right)={\langle G_{ij}^{\text{I}}\left(t\right)\rangle }_{\text{intra}}. \end{array}$$

Here the average is taken over all intrapopulation synapses. Using *G*^intra^(*t*) in Equation (8) yields the expected rate of weight change for intrapopulation synapses

(14) $$\begin{array}{l} J^{\text{intra}}:=\frac{Lf_{\text{RR}}}{M}\overset{\infty }{\underset{-\infty }{\int }}dt^{\prime }\;G^{\text{intra}}\left(t^{\prime }\right)W\left(t^{\prime }-t_{\text{d}}\right). \end{array}$$

Accordingly, we mark quantities related to interpopulation synapses by the suffix “inter”. Neurons that are connected by interpopulation synapses belong to different subpopulations and only receive stimuli simultaneously when both subpopulations are selected for stimulus delivery. Given that one of the subpopulations is already selected, the probability to select the other one as well is (*L* − 1)/(*M* − 1), which yields

(15) $$G^{\text{inter}}(t)={\langle \left(\frac{L-1}{M-1}\right)G_{ij}^{\text{I}}+\left(1-\frac{L-1}{M-1}\right)G_{ij}^{\text{II}}(t)\rangle }_{\text{inter}}.$$

The average is taken over all interpopulation synapses. We introduce the expected rate of weight change for interpopulation synapses as

(16) $$J^{\text{intra}}:=\frac{Lf_{\text{RR}}}{M}\int_{-\infty }^{\infty }dt^{\prime }G^{\text{intra}}(t^{\prime })W(t^{\prime }-t_{\text{d}}).$$

### 2.6. Simulation Details

Numerical integration of the LIF model presented in section 2.1 is performed using an explicit Euler integration scheme with an integration time step of 0.1 ms.

Equation (10) is evaluated numerically using a discretization of the time axis with bin size of 5*10^−3^ ms (Figures 4A,B,E,F,G). Plotted histograms in Figures 4C,D were obtained using a bin size of 0.5 ms. The sum is truncated after *k*_max_ summands, where *k*_max_ is the first integer larger than 500.0ms/τ_Λ_. Convolutions are calculated using the *python* method *numpy.convolve* of *numpy* version 1.16.2.

## 3. Results

To study long-lasting desynchronization by L/M-RR stimulation, we perform numerical simulations of networks of LIF neurons with STDP. For the chosen parameters, a stable synchronized state with strong synaptic connections coexists with a stable desynchronized state with weak connections; see (Kromer et al., 2020). Networks with high initial mean weight approach the synchronized state, while networks with low initial mean weight approach the desynchronized state.

To prepare the network in the synchronized state, we choose a high initial mean weight, 〈*w*〉(*t* = 0) = 0.5. This was realized by selecting 50% of the synapses at random and setting their weights to one, while the other synaptic weights were set to zero. Then, the network is simulated for 500 s in order to reach the synchronous state, see (Kromer et al., 2020) for details.

After preparation, L/M-RR stimulation is delivered for 500 s. Afterward, we continue the simulation for another 1, 000 s to explore potential long-lasting effects of L/M-RR stimulation. To quantify the effect of L/M-RR stimulation, we analyze its acute and long-lasting effects by evaluating the mean synaptic weight 〈*w*〉 and the time-averaged Kuramoto order parameter ρ_Δ_, Equation (6). To quantify acute effects, we evaluate 〈*w*〉 and ρ_Δ_ when the stimulation ceases (*t* = 995 s and Δ = 10 s in Equation 6). Furthermore, to quantify long-lasting effects, 〈*w*〉 and ρ_Δ_ are evaluated 1, 000 s after cessation of stimulation (*t* = 1, 995 s and Δ = 10 s in Equation 6).

**Figure 3:**
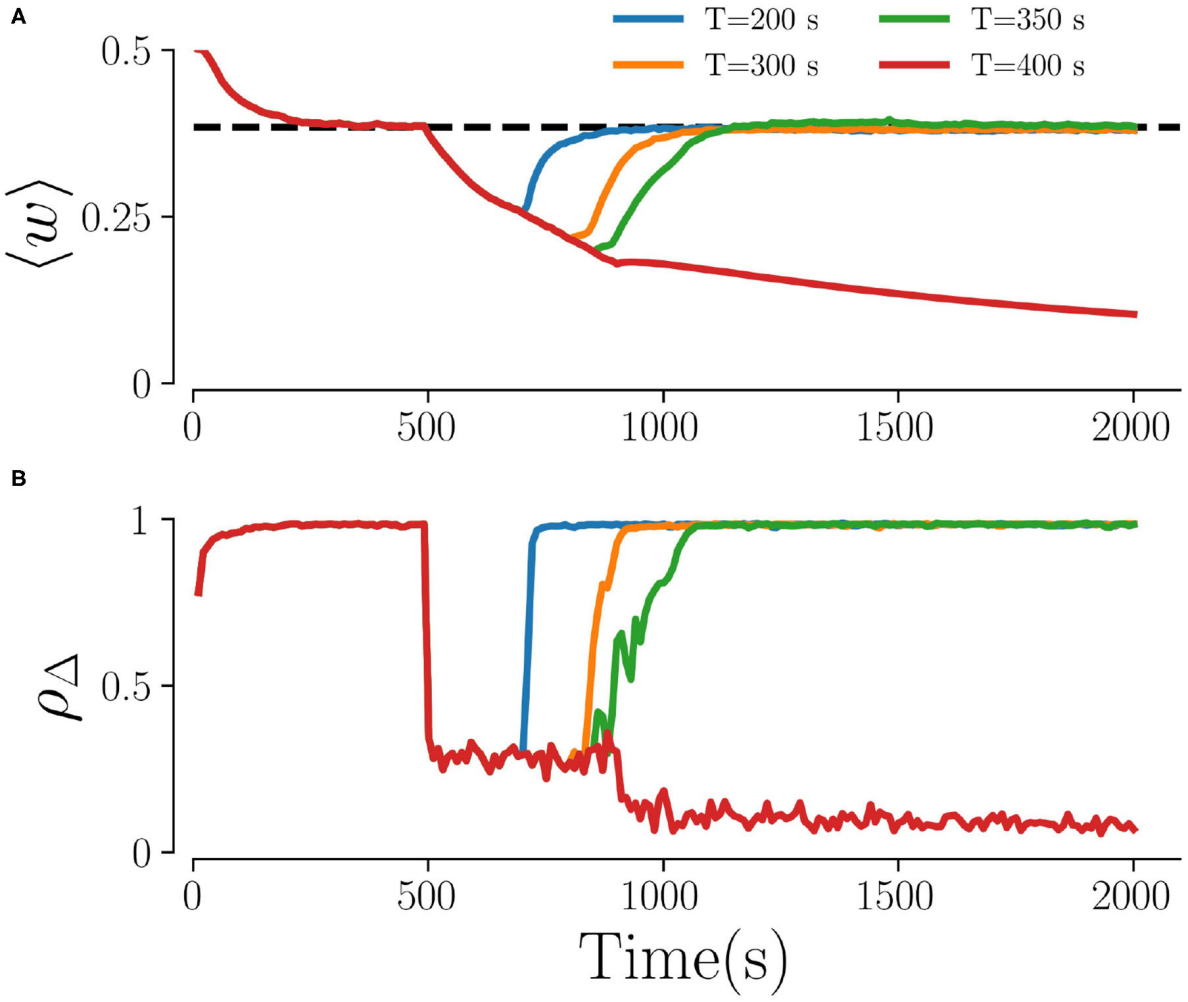
Time traces for different stimulation durations. **(A)** The mean weights, 〈*w*〉, and **(B)** the order parameter, ρ_Δ_, for four different stimulation durations *T*. The dashed line in **(A)** characterizes the weights in the stable synchronized state. Here, *f*_RR_ = 10 Hz, *A*_stim_ = 1, *L* = 5, and *M* = 32. The Kuramoto order parameter in **(B)** is calculated every 10 s by averaging over non-overlapping time windows Δ = 10 s.

Figure 3 shows representative time traces of the mean synaptic weight and the time-averaged Kuramoto order parameter obtained from simulations for different stimulation periods *T*. While the Kuramoto order parameter decreases within seconds after stimulation onset (Figure 3B) the dynamics of the mean synaptic weight is significantly slower, as shown in Figure 3A. As a consequence, the system might not reach the attractor of the stable desynchronized state for short stimulation periods due to insufficient decoupling. Throughout the paper, we fix the stimulation time to 500 s, which turned out to be sufficient for most parts of the parameter space.

Throughout the paper, we time-average the order parameter over Δ = 10 s. Furthermore, results are averaged over three network realizations. We find that results agree qualitatively among these random network realizations. Therefore, and due to the high computational costs, averaging over a large ensemble of network realizations was not performed.

### 3.1. Robust and Long-Lasting Desynchronization for Strong Stimulation

**Figure 4:**
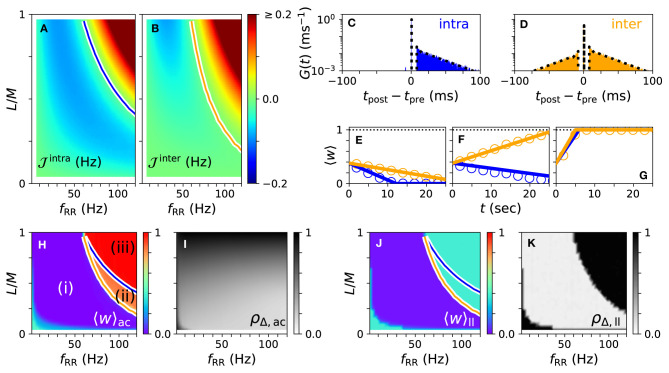
Theoretical predictions for synaptic weight dynamics. **(A,B)** Expected mean rate of weight change for intrapopulation synapses $J^{\text{intra}}$, Equation (14) **(A)**, and interpopulation synapses $J^{\text{inter}}$ **(B)**, Equation (16). Colored curves separate regions with expected strengthening from those with expected weakening of synaptic weights. **(C,D)** Predicted distributions of time lags *G*^intra^(*t*) **(C)**, Equation (13), and *G*^inter^(*t*) **(D)**, Equation (15) (dotted black curves), compared to simulation results (colored histograms). **(E–G)** Theoretical predictions (lines) of mean synaptic weights for intra- (blue) and interpopulation (orange) synapses compared to simulation results (circles) for different stimulation frequencies *f*_RR_ and fractions of simultaneously stimulated subpopulations *L*/*M*. Parameter combinations correspond to the three possible qualitative effects of L/M-RR stimulation: (i) weakening of all synapses **(E)**, (ii) weakening of intrapopulation synapses **(F)** and (iii) strengthening of all synapses **(G)**. **(H–K)** Overall mean synaptic weight **(H,J)** and time-averaged Kuramoto order parameter **(I,K)** as function of *f*_RR_ and *L*/*M*. Acute values **(H,I)** are compared to long-lasting ones **(J,K)**. Colored curves from **(A,B)** are shown to separate parameter regions (i)–(iii) with predicted strengthening and weakening of intra (blue) and interpopulation (orange) synapses, respectively. Parameters: *M* = 32 with *L* = 15 and *f*_RR_ = 60 Hz **(C–E)**; *L* = 15 and *f*_RR_ = 100 Hz **(F)**; and *L* = 25 and *f*_RR_ = 100 Hz **(G)**. We used λ(*t*) = δ(*t*) for theoretical predictions. “ac” and “ll” refer to acute and long-lasting effects, respectively.

First, we consider the case of strong stimulation *A*_stim_ = 1 in which neuronal spikes follow the stimulus pattern. Theoretical predictions for the mean rates of weight changes of intrapopulation synapses, $J^{\text{intra}}$, and interpopulation synapses, $J^{\text{inter}}$, are shown in Figures 4A,B for a wide range of stimulation frequencies and fractions of simultaneously stimulated subpopulations *L*/*M*. Negative rates of weight change, $J^{\text{intra}}<0$, and $J^{\text{inter}}<0$, indicate a weakening of corresponding synapses during stimulation.

A detailed comparison between simulation results and theory is presented in Figures 4C–G. We find an excellent quantitative agreement for the distributions of time lags, Figures 4C,D, and trajectories of the mean synaptic weights of both intra- and interpopulation synapses after the onset of stimulation, Figures 4E–G.

In Figures 4H,I, we show results for the mean synaptic weight, Figure 4H, and the Kuramoto order parameter, Equation (6), Figure 4I, shortly before stimulation ceases. Our theory separates the parameter space into three regions: (i) a region with $J^{\text{intra}}<0$ and $J^{\text{inter}}<0$ where L/M-RR stimulation decouples all neurons, (ii) a region where only intrapopulation synapses are weakened ($J^{\text{intra}}<0$ and $J^{\text{inter}}>0$), and (iii) a region where all synapses are strengthened ($J^{\text{intra}}>0$ and $J^{\text{inter}}>0$), see Figure 4H. Our simulation results show that in region (i) L/M-RR stimulation causes long-lasting desynchronization, while the system returns to the synchronized state in regions (ii) and (iii), see Figures 4J,K.

Next, we analyze the impact of the fraction of simultaneously active subpopulations *L*/*M* on long-lasting effects. Two qualitatively different frequency ranges can be found in Figures 4A,B. For low stimulation frequencies, *f*_RR_ < 60 Hz, stimulation improves as *L*/*M* increases. In contrast, most pronounced long-lasting effects for high stimulation frequencies, *f*_RR_ > 60 Hz, are observed at finite values of *L*/*M*.

### 3.2. Moderate Stimulation Yields Most-Pronounced Long-Lasting Effects

**Figure 5:**
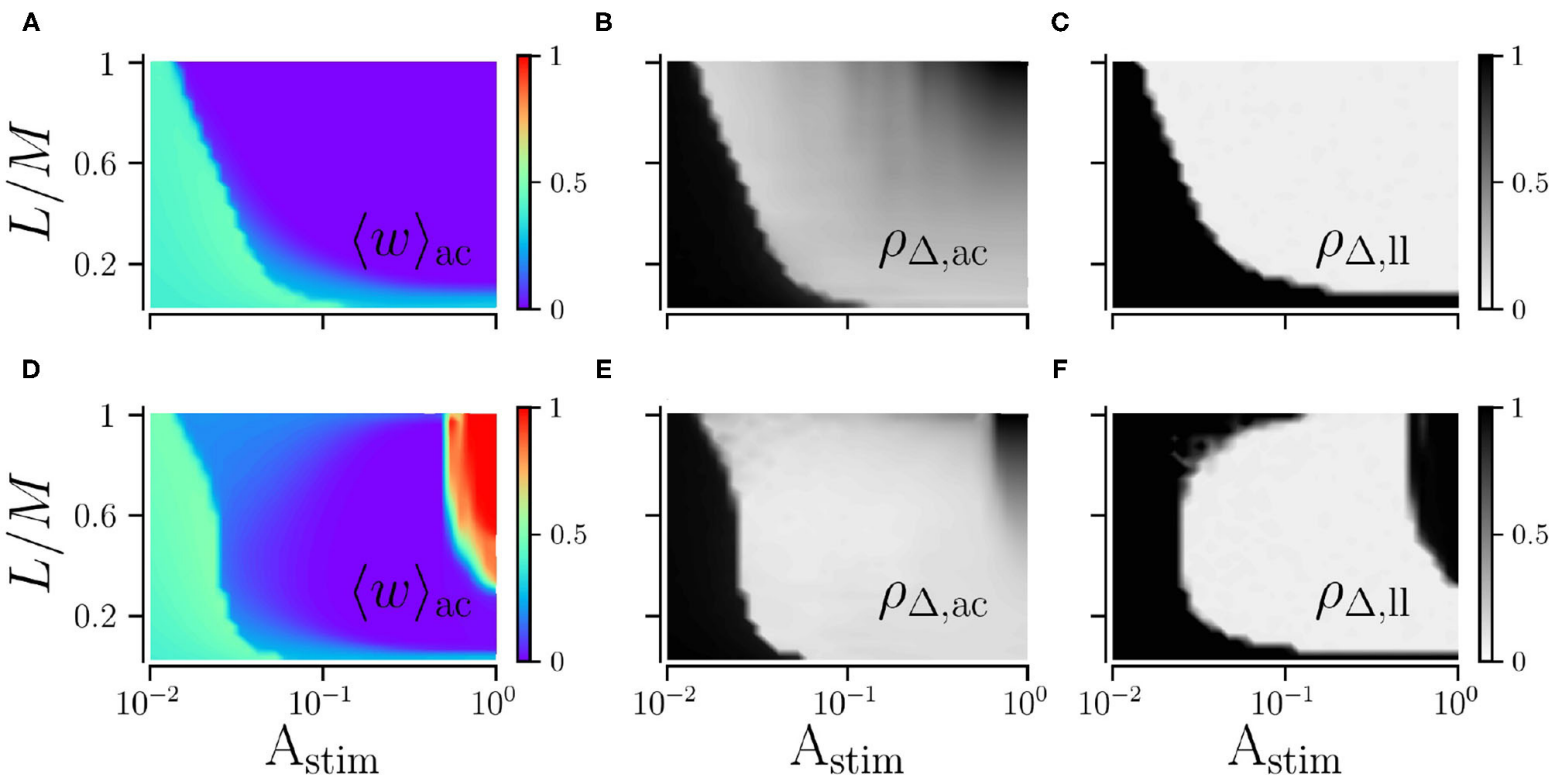
Acute and long-lasting effects of L/M-RR stimulation. Effects of low-frequency **(A,B)** and high-frequency stimulation **(D,E)** shortly before the cessation of stimulation. Mean synaptic weight **(A,D)** and time-averaged Kuramoto order parameter **(B,E)** are shown. Additionally, we show results for the time-averaged Kuramoto order parameter evaluated 1, 000 s after cessation of stimulation in **(C)** (low-frequency stimulation) and **F** (high-frequency stimulation) to quantify long-lasting desynchronization effects. Parameters: *f*_RR_ = 30 Hz (low-frequency stimulation, **A–C**) and *f*_RR_ = 100 Hz (high-frequency stimulation, **D–F**). Stimulation duration is 500 s and *M* = 32. Results were averaged over three network realizations. The acute effects are measured shortly before cessation of stimulation and long-lasting effects 1, 000 s after cessation of stimulation. “ac” and “ll” refer to acute and long-lasting effects, respectively.

We study the impact of the stimulation amplitude *A*_stim_ on acute and long-lasting effects of L/M-RR stimulation for low and high stimulation frequencies. Results are shown in Figure 5. We find that the long-lasting effects of low-frequency stimulation differ significantly from those of high-frequency stimulation. For low stimulation frequencies, stronger stimulation yields better results, see Figures 5A–C. Furthermore, for a smaller fraction of *L*/*M*, stronger stimulation is required to get sustained long-lasting effects. In contrast, for high stimulation frequencies, moderate stimulation yields pronounced long-lasting desynchronization, see Figure 5F, while weak and strong stimulation does not induce long-lasting desynchronization for a wide range of fractions *L*/*M*. Remarkably, we also find that the system returns to the synchronized state after L/M-RR stimulation with very low and very high ratios *L*/*M*, Figure 5F. Therefore, L/M-RR stimulation is most efficient for moderate stimulation amplitudes and intermediate ratios of simultaneously stimulated neuronal subpopulations.

**Figure 6:**
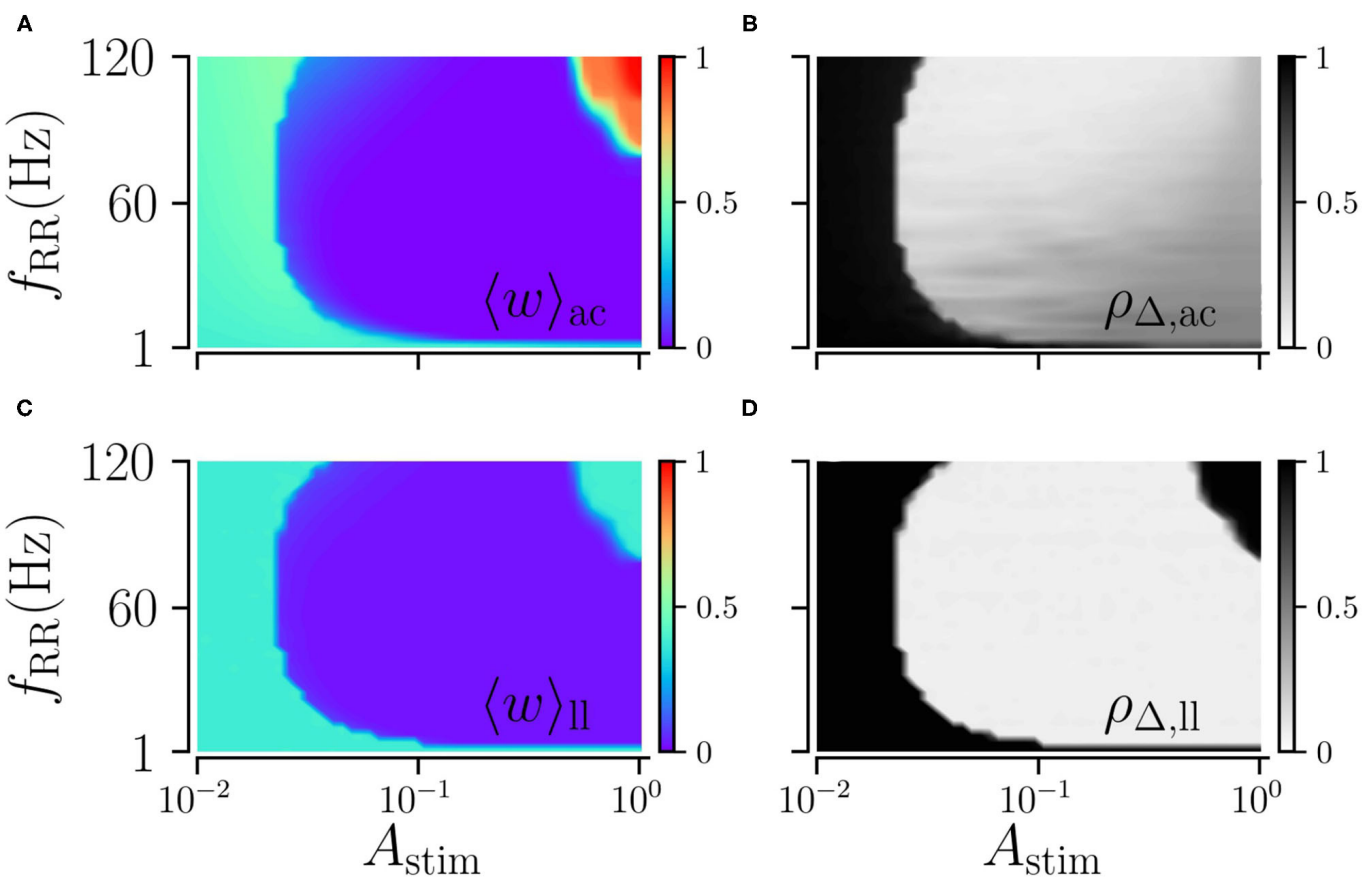
Acute and long-lasting effects of L/M-RR stimulation. Acute mean weight **(A)** and time-averaged Kuramoto order parameter **(B)** as function of stimulation strength *A*_stim_ and stimulation frequency *f*_RR_. **(C,D)** Same as **(A,B)** but evaluated 1, 000 s after cessation of stimulation. Parameters: stimulation is delivered for 500 s, *L* = 16, and *M* = 32. “ac” and “ll” refer to acute and long-lasting effects, respectively.

Next, we explore the dependence of long-lasting effects on the stimulation amplitude, *A*_stim_ ≤ 1, and the stimulation frequency, *f*_RR_. We find that weak and moderate stimulation are most efficient for intermediate stimulation frequencies, as shown in Figure 6. In contrast, strong stimulation only entails long-lasting desynchronization if applied at low stimulation frequencies.

### 3.3. Stimulation With Low Spatial Resolution Performs Better at Low Stimulation Amplitudes

**Figure 7:**
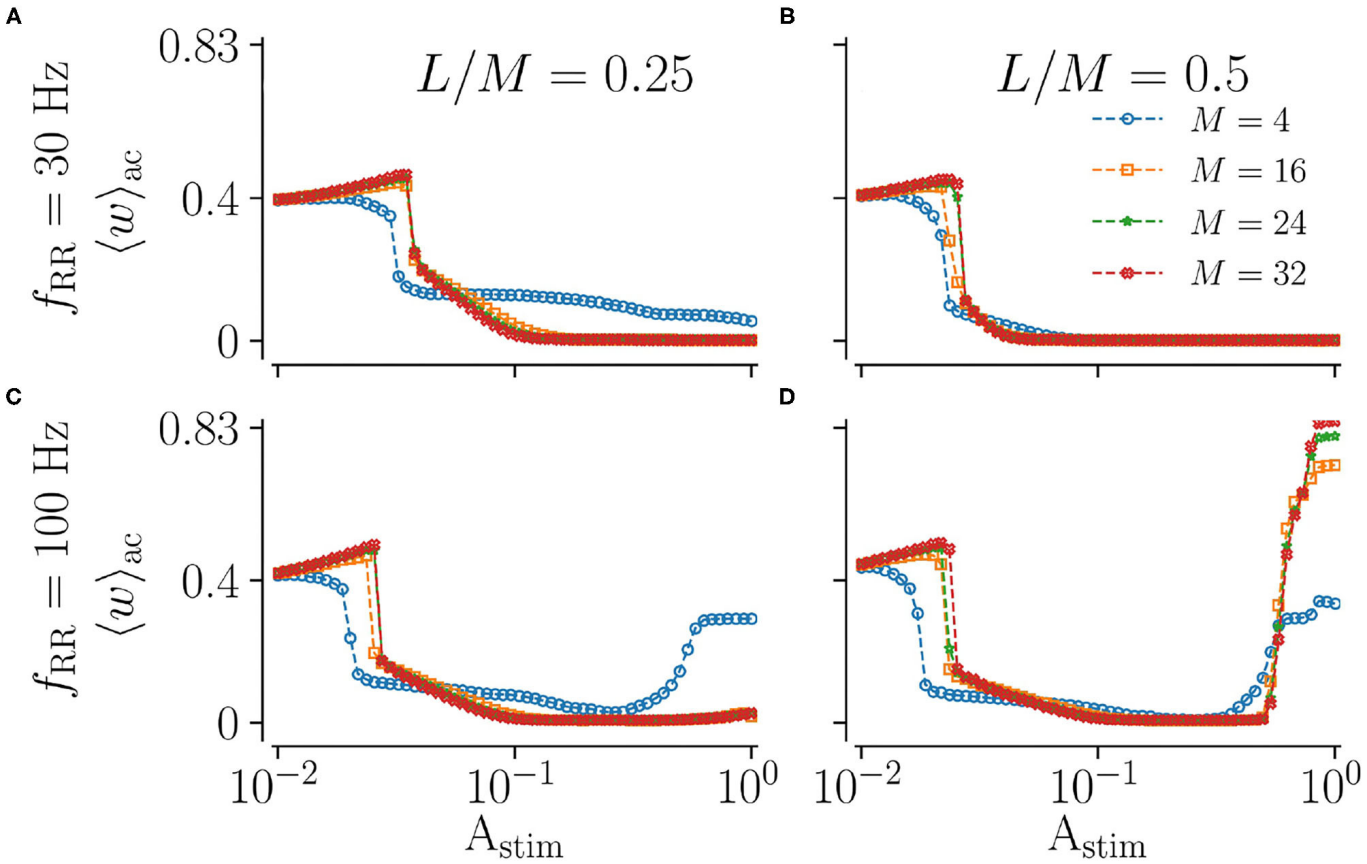
Acute effect of L/M-RR stimulation on the mean synaptic weight for different values of total numbers of subpopulation, *M*. Results for the mean synaptic weight shortly before cessation of stimulation for low-frequency **(A,B)** and high-frequency **(C,D)** stimulation are shown. Colors indicate different total numbers of subpopulations, *M*. Columns contain results for different fractions of simultaneously stimulated neuronal subpopulations *L*/*M*. Parameters: Stimulation is delivered for 500 s. “ac” and “ll” refer to acute and long-lasting effects, respectively.

Next, we vary the spatial resolution *M*, scaling the distance between adjacent stimulation sites (Figure 2). To this end, we fix the fraction of simultaneously stimulated subpopulations *L*/*M* and consider different spatial resolutions *M*. Results for low and high stimulation frequencies *f*_RR_ are shown in Figure 7. We find that the spatial resolution strongly impacts weight reduction for weak and strong stimulation. Low resolutions (small *M*) seem to be advantageous for weak stimulation, where smaller amplitudes are sufficient to achieve a pronounced weakening of synapses. In contrast, a large *M* leads to more synaptic weakening for moderate stimulation and a low fraction of simultaneously stimulated sites (Figures 7A,C). Results for strong low and high-frequency stimulation differ significantly. For low stimulation frequencies, we observe pronounced decoupling for all considered spatial resolutions. Contrastingly, for high-frequency stimulation, the mean synaptic weight shortly before stimulation ceases possesses a complex dependence on the fraction of simultaneously activated sites and the spatial resolution. Here, low resolutions result in higher mean weights for small fractions (Figure 7C). For high fractions, however, high spatial resolution may even strengthen synaptic weights, rendering stimulation not suitable for inducing long-lasting desynchronization. This can be seen in Figure 7D, where strong high-frequency stimulation results in large values of the mean synaptic weight. The latter increases with increasing resolution *M*. Hence, stimulation approaches with low spatial resolution may be advantageous for weak and very strong stimulation.

## 4. Discussion

We studied desynchronization in networks of leaky integrate-and-fire (LIF) neurons with spike-timing dependent plasticity (STDP) by Random Reset (RR) stimulation, a decoupling stimulation technique (Kromer and Tass, 2020). RR stimulation was designed to specifically target synaptic weights and drive plastic neuronal networks into the attractor of a stable desynchronized state with weak synaptic connections. This stabilizes desynchronized activity after cessation of stimulation and may lead to long-lasting desynchronization effects (Kromer and Tass, 2020). The original RR stimulation paradigm suggests the delivery of temporally and spatially randomized stimulus patterns. To realize spatial randomization, (Kromer and Tass) delivered each stimulus to 50% of the neurons. These neurons were selected at random, irrespective of their locations in space and their distance to stimulation contacts (Kromer and Tass, 2020). In a DBS setup, however, such a microscopic selection process is not possible due to limited spatial resolution. There, each stimulus affects a finite tissue volume, while the approach of Kromer and Tass (2020) would require that even nearby neurons can be stimulated independently.

Here, we present a version of RR stimulation that copes with limited spatial resolution. Specifically, each stimulus is delivered to a spatially coherent group of *L* out of *M* randomly selected stimulation sites, denoted as L/M-RR stimulation. This setup mimics the delivery of DBS through modern segmented lead electrodes with multiple stimulation contacts (Steigerwald et al., 2016). L/M-RR stimulation does not require single-neuron stimulation as the approach presented in Kromer and Tass (2020), where stimuli were administered to 50% of the neurons that were randomly selected without considering their distribution and location in space.

In order to analyze the performance of L/M-RR stimulation, we apply a recently developed theoretical framework to predict the mean rate of the stimulation-induced reshaping of intrapopulation and interpopulation synapses. The latter connect neurons in the same and different subpopulations, respectively (Kromer and Tass, 2020). We find an excellent agreement between theoretical predictions and numerical simulations for strong stimulation amplitudes, *A*_stim_ ≈ 1, where neuronal spiking follows the stimulus pattern.

L/M-RR stimulation causes parameter-robust long-lasting desynchronization effects. We find stimulation-induced decoupling and related long-lasting desynchronization in the major part of the parameter space spanned by the stimulation frequency and the fraction of simultaneously stimulated subpopulations *L*/*M* (Figure 4). Only for high stimulation frequencies and large fractions, L/M-RR stimulation does not entail long-lasting desynchronization, i.e., it is ineffective. Here, stimuli are delivered at a high pace, which causes high neuronal firing rates and short time lags between post- and presynaptic spiking events. As these time lags become of the order of the STDP decay time for LTP, τ_+_, synaptic weights start increasing, and stimulation becomes ineffective. This effect causes a qualitative difference between low and high frequency stimulation that is well-described by our theory. Based on our results, we would expect a qualitatively similar outcome for the original RR stimulation protocol presented in Kromer and Tass (2020). There, the percentage of simultaneously stimulated neurons might be comparable to the fraction *L*/*M* of simultaneously stimulated subpopulations in the present paper. However, this percentage was not varied systematically in Kromer and Tass (2020). We further expect our results to be robust with respect to the specific choice of the distribution of interstimulus intervals, Equation (7), as long as the resulting distributions of time lags lead to a sufficiently negative rate of weight change, Equation (8).

Qualitative differences between low- and high-frequency stimulation, observed for strong stimulation, are also present for moderate stimulation amplitudes. While strong high-frequency stimulation is ineffective, at moderate stimulation amplitudes, it leads to long-lasing effects. This is because neurons do not spike in response to stimuli of moderate strength that are delivered shortly after spiking. This leads to longer time lags between post- and presynaptic spikes, which reduces the contribution of LTP to the synaptic weight dynamics and supports stimulation-induced decoupling. This effect leads to a trade-off between strong and weak stimulation. For the former, stimuli hardly impact neuronal spiking, whereas the latter induces short time lags that lead to LTP. As a consequence, L/M-RR stimulation performs best at intermediate stimulation amplitudes. This trade-off differs significantly from those presented in earlier studies reporting optimal performance of coordinated reset (CR) stimulation at intermediate stimulation amplitudes (Lysyansky et al., 2011; Popovych and Tass, 2012; Ebert et al., 2014; Zeitler and Tass, 2015). These studies considered spatial stimulation profiles, where strong stimulation affects larger neuronal populations (Butson and McIntyre, 2008). The latter reduces the decoupling effects of CR stimulation. In contrast, the performance of L/M-RR stimulation becomes worse at strong stimulation amplitudes due to an increased contribution of LTP to the synaptic weight dynamics, due to shorter time lags.

That high stimulation frequencies can lead to qualitatively different synaptic weight dynamics was also observed in our recent study on multisite CR stimulation (Kromer et al., 2020). There, high stimulation frequencies could lead to time lags between post- and presynaptic spikes that are even shorter than the synaptic transmission delay. This led to highly non-linear weight dynamics as a function of the stimulation frequency and the number of stimulation sites, i.e., the spatial resolution (Kromer et al., 2020). In contrast, in the present paper we limited the minimum interstimulus interval to τ_Λ_ > *t*_d_. This ensures non-overlapping stimuli. Therefore, observed time lags are always larger than the delay time. Nevertheless, we observe synaptic strengthening for strong high-frequency stimulation and large numbers of stimulation sites, which is in accordance with the results presented in Kromer et al. (2020) for high-frequency CR stimulation. By the way, very similar results were obtained for a minimum interstimulus interval τ_Λ_ = 1/250 s.

An interesting question is to which extent our results for strong high-frequency L/M-RR stimulation translate to HF DBS. It is widely observed that symptoms return shortly after cessation of HF DBS therapy in Parkinson's patients (Temperli et al., 2003); hence HF DBS may not stabilize physiological activity after cessation of stimulation. RR stimulation represents a temporally and spatially randomized stimulation approach (Kromer and Tass, 2020). This raises the question of whether long-lasting effects may be caused by sufficient randomization of HF DBS. So far, temporally randomized versions of HF DBS were analyzed in a few experimental studies; however, there are mixed results on its efficacy. Furthermore, to our knowledge, all studies were intraoperative and limited to acute effects during stimulation. In Dorval et al. (2010), a randomized HF DBS pattern was used to treat bradykinesia in PD patients. In their study, interpulse intervals were distributed according to a gamma distribution. The authors hypothesized that regular HF DBS leads to symptom alleviation by reducing the firing irregularities in the basal ganglia; they argue that randomized HF DBS fails to regularize the firing and is therefore inferior to regular HF DBS. Birdno et al. (2012) and Brocker et al. (2013) considered five different irregular types of HF DBS. Two deterministic patterns in which a regular pulse train was either interrupted by periods of silence or periods of high-frequency bursts (Birdno et al., 2012; Brocker et al., (2013); and three randomized pulse trains in which interpulse intervals where distribution according to log-uniform distributions of two different widths (Birdno et al., 2012; Brocker et al., 2013) and according to a bimodal distribution where the inverse interpulse intervals were either shorter or longer than the range of therapeutic frequencies (Birdno et al., 2012). Birdno et al. (2012) found that irregular HF DBS is inferior to the regular one in treating tremor. They argue that pathological activity may propagate during long interpulse intervals. In contrast, Brocker et al. (2013) studied the performance of PD patients in a simple motor task (finger tapping) and reported improved performance during irregular HF DBS. Moreover, their computational model showed that these randomized DBS patterns significantly suppressed beta band power. However, as acknowledged by the authors, the intraoperative setting of their clinical trials limits the duration of the experiment. Therefore, possible effects might not fully develop, and the therapeutic effects of irregular HF DBS might be underestimated.

It is unclear whether experimental results on irregular HF DBS apply to the L/M-RR stimulation protocol suggested in the present study. Furthermore, the L/M-RR stimulation differs in two ways from the studies on irregular DBS. First, L/M-RR stimulation targets pathological connectivity rather than pathological neuronal activity. Long-lasting changes due to randomized HF DBS, however, have yet to be studied. As shown in Figure 3, L/M-RR stimulation needs to be administered for a sufficient amount of time to drive the network into the attractor of the stable desynchronized state. For too short stimulation time, the full potential of long-lasting effects might not be released (Figure 3). Furthermore, as pointed out by our theory for strong stimulation, the stimulation-induced weight dynamics is closely related to the statistics of the interstimulus interval, see Equation (8). Whether the irregular HF DBS protocols studied in Dorval et al. (2010), Birdno et al. (2012), and Brocker et al. (2013) cause a reduction of synaptic weights further depends on the underlying plasticity mechanism. Once the latter has been explored, Equation (8) might be used to predict the potential long-lasting outcome. Second, L/M-RR stimulation combines temporal and spatial randomization, while only temporally randomized HF DBS was considered. In fact, we find that high-frequency L/M-RR stimulation is ineffective for large fractions of simultaneously activated stimulation sites, while low fractions result in pronounced long-lasting effects (Figures 5D–F).

The fraction of simultaneously activated subpopulations *L*/*M* controls the degree of spatial randomization. A fraction of *L*/*M* = 1 corresponds to spatially regular single-site stimulation, while low fractions result in a high degree of spatial randomization. This fraction also impacts the frequency at which individual subpopulations receive stimuli. In particular, neurons receive stimuli at higher rates if this fraction is increased for fixed stimulation frequency. However, our computational results show that this only improves long-lasting effects for low stimulation frequencies (Figures 5A,B). In contrast, increasing this fraction yields worse performance for high-frequency stimulation (Figures 5C,D). Improvement of long-lasting effects at low stimulation frequencies results from the so-called *decoupling through synchrony*, which occurs for asymmetric Hebbian plasticity functions if the distribution of spike times within collective spiking events becomes narrow compared to axonal delays (Lubenov and Siapas, 2008; Knoblauch et al., 2012). In the present paper, we use short stimuli that cause such narrow distributions of spike times during collective spiking events (Kromer and Tass, 2020). This supports decoupling when stimuli are simultaneously delivered to a large number of subpopulations, i.e., *L*/*M* ≈ 1, see Figure 5A. For high stimulation frequencies, however, this effect is balanced by LTP due to short time lags, as described in detail above. Therefore, high-frequency stimulation is ineffective for *L*/*M* ≈ 1. As a result, spatial randomization resulting from intermediate fractions *L*/*M* increases the robustness of long-lasting desynchronization by L/M-RR stimulation with respect to changes in the stimulation frequency.

Of particular interest with respect to a possible implementation of L/M-RR stimulation using a DBS setup is the impact of the number of stimulation sites, represented by the spatial resolution *M*. Commonly used DBS electrodes possess 4–8 stimulation contacts that are arranged equidistantly along the electrode axes (Gielen, 2001; Butson and McIntyre, 2005). Recently, Steigerwald et al. (2019) presented an electrode with up to 32 stimulation contacts that can be activated independently. For strong stimulation, the impact of the number of stimulation sites *M* can be analyzed theoretically. Our theory predicts that the dynamics of intrapopulation weights solely depends on the fraction of simultaneously activated subpopulations *L*/*M*, see Equations (9), (10), and (13). In contrast, the probability of simultaneous activation of different subpopulations, and therefore the dynamics of interpopulation weights depends on (*L* − 1)/(*M* − 1), see Equations (15) and (16). Thus, changes of the spatial resolution, *M*, while keeping the fraction *L*/*M* constant, only affect the dynamics of interpopulation weights. As a consequence, after sufficiently long stimulation different mean synaptic weights can be attained, depending on the combination of signs of the mean rates of weight change $J^{\text{intra}}$ and $J^{\text{inter}}$, i.e., whether stimulation reduces all weights ($J^{\text{intra}}<0$ and $J^{\text{inter}}<0$), increases all weights ($J^{\text{intra}}>0$ and $J^{\text{inter}}>0$), or whether only one type of weights is reduced while the other one increases. While this determines the outcome of strong stimulation, our simulation results provide evidence that low spatial resolution is favorable for weak stimulation, as shown in Figure 7. We hypothesize that this is because simultaneously stimulated subpopulations consist of bigger localized groups of neurons and such groups are more likely to follow the applied stimulus pattern. Since sufficient decoupling is already achieved for weak stimulation with low spatial resolution, L/M-RR stimulation may be suitable for implementation in a conventional DBS setup. There, weak stimulation reduces the risk of side-effects (Rodriguez-Oroz et al., 2005). Furthermore, low spatial resolution, i.e., a smaller number of stimulation sites, is advantageous because it allows the usage of common DBS electrodes and reduces the time for preparation and parameter tuning. In addition, using a smaller number of simulation sites may increase feasibility given the anatomical and topological constraints of currently available chronically implantable depth electrodes and the related consequences on therapeutic effects and side effects (Volkmann et al., 2006; Krauss et al., 2020).

So far, segmented electrodes were mainly used intraoperatively for directional HF DBS stimulation. During directional DBS, the current flow can be directed in both the vertical and in the horizontal plane by activation of a select number of individual contacts. This results in high spatial selectivity (Contarino et al., 2014; Pollo et al., 2014; Steigerwald et al., 2019). These intraoperative studies suggest that directional DBS may lower the threshold current for beneficial HF DBS (Contarino et al., 2014; Pollo et al., 2014). However, the full potential of segmented electrodes for multisite stimulation methods such as CR or L/M-RR stimulation is yet to be explored. In the present paper, we suggest L/M-RR stimulation as one way to realize the recently developed RR stimulation paradigm by means of segmented electrodes. Our theoretical and computational results indicate that separate stimulation of a large number of neuronal subpopulations, which may correspond to a large number of stimulation contacts, improves long-lasting desynchronization by L/M-RR stimulation for low stimulation frequencies, Figure 7A.

In future studies, we anticipate exploring L/M-RR stimulation in a detailed biophysical model of the subthalamic nucleus, a major target region for therapeutic HF DBS stimulation in Parkinson's patients (Benabid et al., 1991) using more detailed models of stimulation contacts accounting for spatial current profiles. Furthermore, we plan to use large-scale neuronal network models, to study how STN stimulation affects interactions between different nuclei in the basal ganglia region. We hope that our encouraging results motivate experimental and preclinical studies on RR stimulation as a potential treatment for neurological disorders that exploits recently developed segmented electrodes (Steigerwald et al., 2019).

## Data Availability Statement

The original contributions presented in the study are included in the article/supplementary materials, further inquiries can be directed to the corresponding author/s.

## Author Contributions

AKN, JK, and PT conceived the idea, designed the study, interpreted the results, and wrote the manuscript. AKN performed the numerical simulations and analysis. JK developed the theory. All authors contributed to the article and approved the submitted version.

## Conflict of Interest

PT works as consultant for Boston Scientific Neuromodulation. JK and PT are co-inventors of a patent for random reset stimulation owned by Stanford University. The remaining author declares that the research was conducted in the absence of any commercial or financial relationships that could be construed as a potential conflict of interest.
